# Chromatin remodeler Activity-Dependent Neuroprotective Protein (ADNP) contributes to syndromic autism

**DOI:** 10.1186/s13148-023-01450-8

**Published:** 2023-03-21

**Authors:** Claudio Peter D’Incal, Kirsten Esther Van Rossem, Kevin De Man, Anthony Konings, Anke Van Dijck, Ludovico Rizzuti, Alessandro Vitriolo, Giuseppe Testa, Illana Gozes, Wim Vanden Berghe, R. Frank Kooy

**Affiliations:** 1 Department of Medical Genetics, University of Antwerp, Prins Boudewijnlaan 43/6, 2650 Edegem, Belgium; 2 Protein Chemistry, Proteomics and Epigenetic Signaling (PPES), Epigenetic Signaling Lab (PPES), Department of Biomedical Sciences, University of Antwerp, Universiteitsplein 1, 2610 Wilrijk, Belgium; 3 High Definition Disease Modelling Lab, Stem Cell and Organoid Epigenetics, IEO, European Institute of Oncology, IRCCS, 20141 Milan, Italy; 4 Department of Oncology and Hemato-Oncology, University of Milan, 20122 Milan, Italy; 5 Human Technopole, V. Le Rita Levi-Montalcini, 1, 20157 Milan, Italy; 6 Elton Laboratory for Molecular Neuroendocrinology, Department of Human Molecular Genetics and Biochemistry, Faculty of Medicine, Adams Super Center for Brain Studies and Sagol School of Neuroscience, Tel Aviv University, Sackler School of Medicine, 727, 69978 Tel Aviv, Israel

**Keywords:** Helsmoortel–Van der Aa syndrome, Activity-Dependent Neuroprotective Protein, ADNP, Chromatin remodeler, Autism, intellectual disability, neurodevelopmental disorder, cancer

## Abstract

**Background:**

Individuals affected with autism often suffer additional co-morbidities such as intellectual disability. The genes contributing to autism cluster on a relatively limited number of cellular pathways, including chromatin remodeling. However, limited information is available on how mutations in single genes can result in such pleiotropic clinical features in affected individuals. In this review, we summarize available information on one of the most frequently mutated genes in syndromic autism the Activity-Dependent Neuroprotective Protein (ADNP).

**Results:**

Heterozygous and predicted loss-of-function ADNP mutations in individuals inevitably result in the clinical presentation with the Helsmoortel–Van der Aa syndrome, a frequent form of syndromic autism. ADNP, a zinc finger DNA-binding protein has a role in chromatin remodeling: The protein is associated with the pericentromeric protein HP1, the SWI/SNF core complex protein BRG1, and other members of this chromatin remodeling complex and, in murine stem cells, with the chromodomain helicase CHD4 in a ChAHP complex. ADNP has recently been shown to possess R-loop processing activity. In addition, many additional functions, for instance, in association with cytoskeletal proteins have been linked to ADNP.

**Conclusions:**

We here present an integrated evaluation of all current aspects of gene function and evaluate how abnormalities in chromatin remodeling might relate to the pleiotropic clinical presentation in individual“s” with Helsmoortel–Van der Aa syndrome.

## Introduction

Autism is a devastating condition, diagnosed in early childhood and characterized by qualitative impairments in social interaction and communication skills, accompanied by repetitive and stereotypic behaviors and interests [1]. Interestingly, recent advances in next-generation sequencing allowed large-scale exome sequencing initiatives revealing identification of a large series of genes involved in the disorder [2–4]. While collectively common, mutations in individual genes are rare and, in many cases, only a handful of patients with mutations in any specific gene are identified. Despite the genetic heterogeneity, the genes involved in autism converge on a limited number of biological pathways, including chromatin remodeling [5–7]. Genes involved in chromatin remodeling essentially encode for proteins which have a catalytic function in installing posttranslational histone modifications and DNA modifications (writers), removing such modifications (erasers) or have chromatin remodeling activity (remodelers) [8]. In this review, we discuss the multiple chromatin remodeling properties of the Activity-Dependent Neuroprotective Protein (ADNP) [9]. The *ADNP* gene has been found to be mutated in significant percentage of patients diagnosed with syndromic autism or intellectual disability and is one of its more frequent genetic causes [2–4, 10]. With the expansion of recent studies on the *ADNP* gene and its role in development, a comprehensive review of the function with emphasis on chromatin remodeling is warranted. Here, we provide an overview of what is known about the *ADNP* gene function since its discovery in 1999 till today.

## ADNP is an embryonic gene essential for brain formation

The *ADNP* gene was first discovered as a vasoactive intestinal peptide (VIP)-responsive gene [9]. VIP is active during embryonic development and prevents neuronal cell death by inducing secretion of glia-derived survival-promoting factors. ADNP exerts a critical neuroprotective function, being essential for neural tube formation [11] and modulating its own gene expression [12]. The biological role of ADNP was associated with essential functions such as organogenesis of the developing embryo and proper brain formation [11]. Initial observations of *Adnp* haploinsufficient mice suggested a critical role in behavior with a major impact on cognitive function [13].

**Fig. 1:**
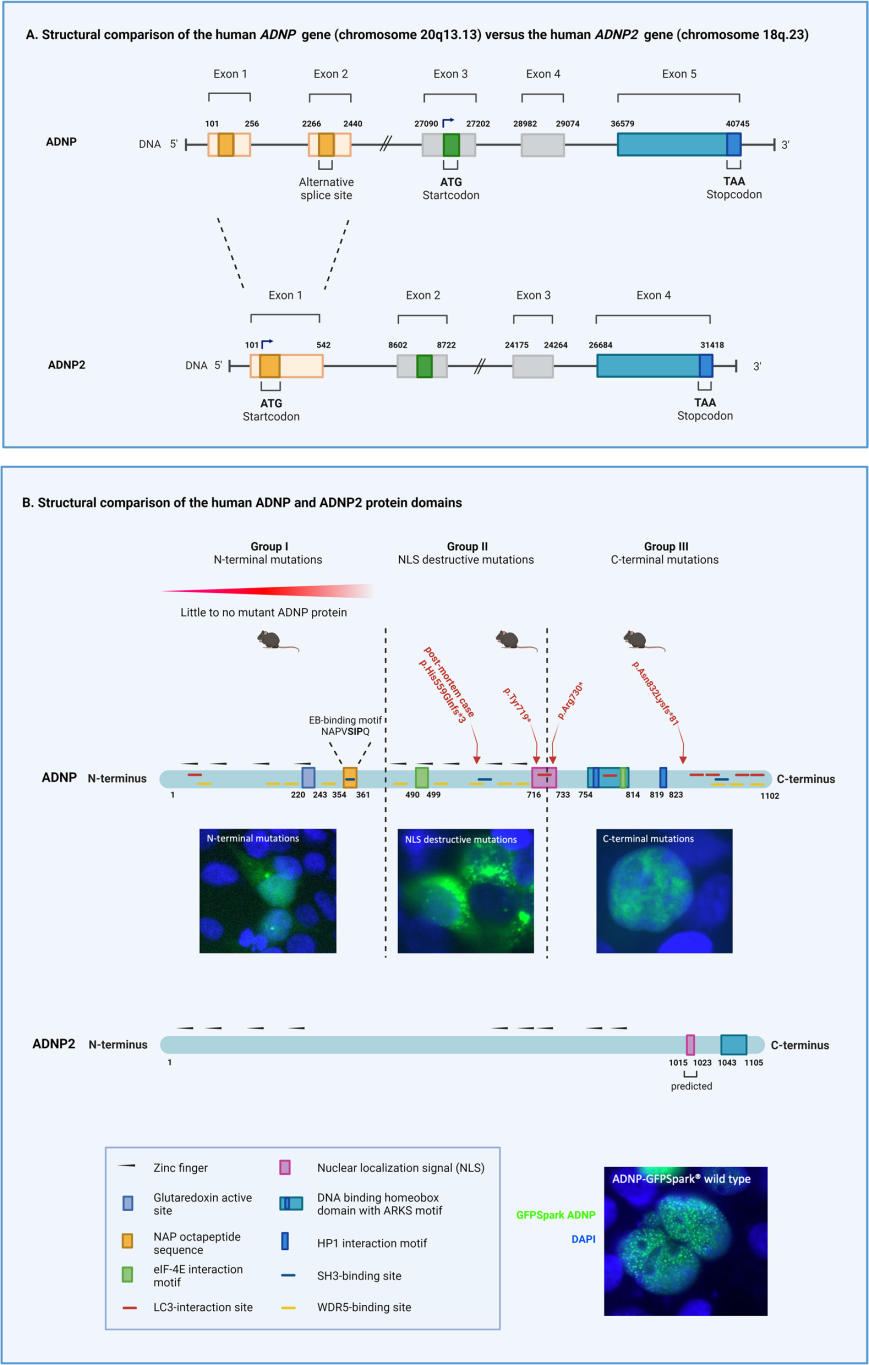
Structural comparison of the ADNP and ADNP2 gene structure and functional protein domains.** (A)** The *ADNP* gene contains five exons of which only the last three are translated (https://www.ensembl.org/). The *ADNP2* gene contains only four exons. The 5’UTR of ADNP2 corresponds with exons 1 and 2 of ADNP. The 3’UTR is comprised of a part of exon 4, correlating to exon 5 of ADNP. ATG, start codon; TAA, stop codon. **(B)** The relative positions of the ADNP nine zinc fingers (lines) together with the glutaredoxin active site, NAP sequence, eIF-4E interaction motif, nuclear localization signal (NLS), DNA-binding homeobox domain with ARKS and PxVxL motif are illustrated on the figure. Computational sequence analysis also revealed LC3 interaction sites (MAP1ALC3), SH3-binding sites (SHANK3), and WRD5-binding sites (SIRT1) in ADNP which could be confirmed by direct co-immunoprecipitation experiments. The *ADNP* gene is divided in three mutational classes: N-terminal, perinuclear (NLS destructive), and C-terminal mutations, each of them altering the subcellular localization and expression of the protein. The most recurring and prevalent *ADNP* mutations of the spectrum include the p.Tyr719*, p.Arg730*, and p.Asn832Lysfs*81 with the unique deceased ADNP toddler mutation c.1676Adupl/p.His559Gln*3. The three viable *Adnp* heterozygous mouse strains mimic in part mutation designated to each class of the mutational spectrum, e.g., haploinsufficient mouse accounting for N-terminal mutations, respectively, the p.Tyr718* *Adnp* mouse for the NLS-destroying group of patient mutations, and the frameshift *Adnp* mouse for patients with C-terminal mutations. ADNP2 shows homology to ADNP by the presence of an equal amount of zinc fingers and a DNA-binding homeobox

Spanning about 40 kb of DNA, the *ADNP* gene maps to the chromosomal position chr20q13.13 in the human genome and is comprised of five exons [10, 14]. Several splice variants have been described of which the longest transcript is 6672 bp ([14]; NCBI; *NM_001282531.3*) with the other variants all differ in the 5’ untranslated region (UTR). The last three exons are common to all transcription variants and translated into functional ADNP protein (Fig. 1A). Of note, an antisense transcript *ADNP-AS1* has been annotated that is transcribed starting from ADNP exon 1 in the opposite direction. Whether it has any anti-sense activity remains to be determined. The ADNP protein consists of 1102 amino acids with a calculated molecular mass of 124 kDa [10, 14]. Spanning amino acids 74–686, nine C_2_H_2_-type zinc fingers were identified, to aid the protein in nucleotide binding in companion with the DNA-binding homeobox domain, located over amino acids 754–814. ADNP also encloses a glutaredoxin active site over position 220–243, which could potentially modulate its own DNA-binding activity or other DNA-binding proteins in response to oxidative stress and signal transduction pathways involved in the redox state of the cell [14, 15]. The neuroprotective function of ADNP is attributed to the octapeptide NAP sequence (NAPVSIPQ = Asn-Ala-Pro-Val-Ser-Ile-Pro-Gln) ranging from the sequence 354 to 361 [9, 16–18]. An immunoprecipitation assay demonstrated the interaction between ADNP and eukaryotic translation initiation factor 4E (eIF-4E) by two putative binding motif sequences on the ADNP amino acid sequence 490—499, namely KclYcnyLp and cekYkpgVLL [19]. The presence of the bipartite nuclear localization signal (NLS) spanning amino acids 716–733 accounts for transport to the nucleus [14, 20]. Moreover, ADNP comprises both an alanine–arginine–lysine–serine (ARKS) motif located within the homeobox domain and a proline–valine–leucine (PxVxL) motif at position 819–823, which render ADNP the possibility to interact with heterochromatin protein 1 (HP1) [12, 21]. The ARKS motif functions in the stabilization of the interplay of HP1 and the PxVxL motif [21] **(Fig. ****1****B)**. Together, both motifs are involved in HP1-dependent H3 lysine 9 trimethylation (H3K9me3) association and localization to pericentromeric heterochromatin [21]. In this respect, multiple WD repeat-containing protein 5 (WDR5) that mediate the assembly of histone modification complexes were discovered on ADNP, tying it also to the WDR5-interacting protein Sirtuin 1 (SIRT1) [22]. Moreover, ADNP was also shown to interact with the autophagy-initiating protein microtubule-associated protein 1 light chain 3 (LC3) by direct immunoprecipitation and presents with LC3-binding sites covered all over the protein [23]. Most recently, a linear motif prediction mapped multiple Src homology 3 (SH3) domain-ligand association sites in ADNP, surprisingly one in the NAP sequence, suggesting a regulatory role of the cytoskeleton [24]. Remarkably, these SH3-binding site are essential to the autism-mutated SH3 and multiple ankyrin repeat domains protein 3 (SHANK3) protein. Using actin-immobilized beads, ADNP and SHANK3 were found to be co-immunoprecipitated, thereby linking a functional role of ADNP binding by its NAP domain via the cytoskeleton with SHANK3.

**Fig. 2:**
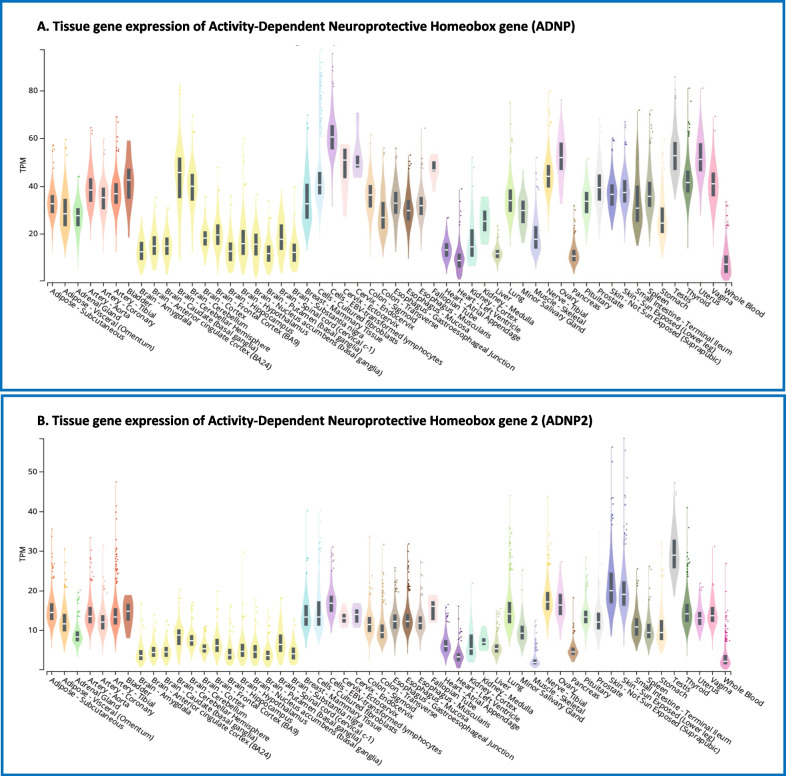
Tissue gene expression of the ADNP and ADNP2 (GTEx Portal). **(A)** Tissue gene expression of ADNP. A high expression of ADNP is reported in brain regions such as the cerebellum and cortex, gastrointestinal tissues, lungs, and reproductive system. A moderate expression is observed in the kidneys, smooth muscles, and soft tissues, while low to absent levels present in cardiac muscles, adipose tissue, liver, and skeletal muscles. **(B)** Tissue gene expression of the ADNP2. A high expression of ADNP2 is observed in brain regions such as the cortex and cerebellum, although lower in comparison with ADNP. Heart, kidneys, and uterine tissues were reported with a moderate expression. Low expression levels are seen in the pancreas, spleen, liver, lungs, and skeletal muscles. TPM, transcripts per million

ADNP is expressed in many tissues with the highest expression in different regions of the human brain, gastrointestinal tissues, lungs, and reproductive system. Moderate expression is observed in the kidneys, smooth muscles, and soft tissues, and low to absent levels in cardiac muscles, adipose tissue, liver, and skeletal muscles [14] (**Fig. ****2****A**). During mouse development, *ADNP* shows a high expression, peaking at embryonic days 9–13, concomitant with neural tube closure, in the entire embryo, while brain expression is sustained into later stages of development [11]. Predominant expression of ADNP is observed in the hippocampus, cortex, and cerebellum [12, 25].

An initial study reported the expression of exogenous ADNP-GFP transfected in HEK293T cells restricted to the nucleus [12]. Human recombinant ADNP was shown to colocalize with defined DAPI-positive foci in the nucleus in vitro. Nuclear DAPI foci have been described to correlate with heterochromatin densities, and the presence of ANDP is such foci were a first lead that the protein can be involved in chromatin remodeling [26]. In the P19 pluripotent teratocarcinoma cell line, ADNP expression was restricted to the nucleus in non-differentiated pluripotent cells and DMSO-differentiated cardiovascular cells [27]. However, following retinoic acid-induced differentiation of P19 pluripotent cells into neuronal cell types, ADNP expression was detectable in the nucleus as well as in the cytoplasm and neuronal extensions in cultured neurites. A nuclear export mechanism has not been formally explained, but is predicted based on some similarity of the leucine-rich nuclear export sequence of chicken engrailed protein 2 (En2) with aa 788–804 ADNP sequence [14, 28], but this observation awaits experimental validation. ADNP expression in other cell types including mouse heart and placenta remained restricted to the nucleus. In rat brain expression was mainly observed in the cytoplasm of neurons and its dendritic extensions signals using immunoreactivity [29]. Occasionally, weak signals indicated a partial nuclear localization in these neurons. In contrast, rat astrocytes showed nuclear and an (almost) absolute lack of cytoplasmatic expression. In rat cortical astrocyte cultures, ADNP was found in the nucleus as well as in the cytoplasm, but also reported in the culture medium, suggesting secretion of the protein [30]. In the astrocyte cytoplasm, ADNP colocalized with tubulin and microtubules, but not with actin filaments, indicating a potential crosstalk of ADNP with the cytoskeleton [30].

In transfection assays in HEK293T cells, wild-type ADNP was reported in the nucleus of transfected [20]. When patient mutations in *ADNP* were modeled in this cellular system, a marked effect on expression and subcellular localization was observed depending on their position in the protein. Mutations at the C-terminal side of the NLS were expressed in the nucleus, similar to wild-type ADNP. Mutations in the central region of ADNP that result in a truncated protein lacking the NLS sequence were mislocalized in the cytoplasm. As mutations approached the N-terminus, ADNP expression was absent or at least undetectable since these short fragments undergo ubiquitination and are prone to proteasomal decay.

Genome editing (CRISPR/Cas9) in N1E-115 neuroblastoma cells to form neuron-like cell lines expressing ADNP-mutant proteins conjugated to GFP indicated distinct cellular phenotypes depending on the mutation location [31]. Truncating mutation close to the N-terminus showing increased neurite numbers in non-differentiated cells followed by increased neurite lengths upon differentiation. In contrast, a mutation destroying the NLS showed decreased cell numbers in non-differentiated cells. Both mutant proteins showed elevated expression in the cytoplasm compared to the non-mutated GFP-ADNP. Reduced nuclear/cytoplasmic boundaries were observed with both mutations, but most notably in the NLS-truncated ADNP-mutant line. Recently, the subcellular localization of Adnp was investigated in somatosensory-derived primary neurons, where Adnp was visualized in the nucleus of undifferentiated neurospheres but showed a remarked cytoplasmic shift in differentiated cortical neurons after neuritogenesis [32]. Moreover, co-immunoprecipitation experiments demonstrated binding of Adnp to several 14-3-3 protein isoforms with a well-known function in nuclear-cytoplasmic protein localization. Also, administration of the 14-3-3 inhibitor difopein restricted Adnp expression solely to the nucleus, thereby indicating that Adnp shuttles to the cytoplasm by the 14-3-3 nucleocytoplasmic shuttling protein.

Together, these findings indicate a developmental, cell, tissue, and mutation-specific expression of ADNP, suggesting a multimodal function of the protein in health and disease.

## ADNP is a highly conserved chordate-specific gene that only shows homology with its paralogue Activity-Dependent Neuroprotective 2 gene (ADNP2)

The original cloning of human ADNP identified a single paralogous sequence in the human genome later named Activity-Dependent Neuroprotective Protein 2 (ADNP2) [14, 33]. The sequence of human *ADNP* and *ADNP2* shows 33% identity and 46% similarity. The genomic structure of the human *ADNP2* gene resembles that of *ADNP*, but contains only 4 exons, lacking a paralogue of the non-coding exon 2 **(Fig. ****1****A)** [14, 34]. The ADNP2 protein is estimated to be 1131 amino acids long with a theoretical molecular weight of 122.8 kDa (https://www.uniprot.org/). Based on the presence of putative zinc fingers and homeobox domain, ADNP2 has a suggested function in cell signaling, cell structure and motility and chromatin remodeling. A monopartite NLS sequence VPFKRQRNE starting from amino acid position 1015–1023 was predicted by the cNLS Mapper motif prediction program [34, 35] (**Fig. ****1****B**). Overall, the expression pattern of ADNP2 resembles that of ADNP, showing a high expression in tissues such as the cortex and cerebellum. Other tissues such as the heart, kidneys, and uterus were reported with a moderate expression. Low expression levels are observed in the testis, pancreas, spleen, liver, lungs, and skeletal muscles [33] (**Fig. ****2****B**). Interestingly, ADNP2 also showed a high expression in utero at E7.5, two days before the expression peak of its paralogue ADNP at E9.5, suggesting a crucial role in development, formation, and function of the brain [11, 33]. In search for ADNP2 function in vivo using a zebrafish model let to the discovery of an evolutionary conserved role for the ADNP protein family, essential for erythropoiesis [34]. Both ADNP and ADNP2 were discovered to regulate *beta-globin,* both interacting with BRG1, a key chromatin remodeling SWI/SNF component, and with ADNP directly interacting with the beta-globin locus control region.

## ADNP in neurodevelopment

*ADNP* mRNA is enriched in the mouse brain (hippocampus, cerebellum), in comparison with peripheral tissues, implicating an important role for ADNP in brain functioning [9]. Mouse embryonic *ADNP* mRNA reaches its maximum expression level at E9.5, when cranial neural tube closure takes place [11]. After E14.5, ADNP expression decreases in the whole embryo but was sustained in the embryonic brain. In *Adnp* knockout (KO) mouse embryos that show lethal defects in brain formation and neural tube closure, *Oct4* expression was upregulated, while *Pax6* expression was downregulated and even absent in the anterior neural plate [11]. *Pax6* plays an important role in the development of the central nervous system and defects in *Pax6* expression can cause, for example, microcephaly [36], which was originally associated with the ADNP-regulator VIP functional deficiency [37]. Further observations looking at *Adnp* knockout embryos suggested generally inhibited development (smaller embryos). In P19-cell-derived neurons, ADNP inhibition resulted in a reduced neurite number [27], paralleling the effect observed following a downregulation of the SWI/SNF (BAF) complex [reviewed in 38]. In line with these observations, *Adnp* KO mESCs were not able to form organized embryoid bodies and showed downregulation of neuroectodermal genes, including *Pax6* and *Nestin*. Differentiated neural progenitor cells (NPC) from *Adnp* KO mESCs showed downregulation of *Nestin*, and only 31% was Pax6 positive, compared to the control NPCs with a 60% *Pax6* positivity rate. At day 19 of differentiation, the *Adnp* KO mESCs showed less neuronal fibers and reduced TuJ1 and GFAP signals. RNA-seq at different time points during differentiation showed that neuroectodermal genes were downregulated, while pluripotency and primitive endodermal markers were upregulated in *Adnp* KO mESCs. These defects could partially be rescued when ADNP expression was restored in an early stage, again stressing the role of ADNP in neural development [39]. Along the same line, the observed ADNP regulation [40] and interaction with components of the Wnt/*β*-catenin pathway [41] further support its critical involvement in neural development [42]. More recently, ADNP was found to interact through its N-terminus with the armadillo domain of *β*-catenin, thereby stabilizing the protein, and protect it from hyperphosphorylation and degradation [41].

## Mutations in ADNP result in Helsmoortel–Van der Aa syndrome (HVDAS)

Genome-wide whole-exome studies in patients with autism co-morbid with intellectual disability (ID) revealed an excess of truncating de novo mutations in the *ADNP* gene is (*p* = 0.001852, odds ratio = 13.24668, one—sided Fisher’s exact test) [10, 43]. In an initial study, 10 individuals with a syndromic version of autism were described. In subsequent screening studies using whole-exome or molecular inversion probes in large cohorts of patients with ID and or autism in large cohorts of thousands of patients, *ADNP* is consequently among the most frequently mutated genes observed [2–4, 44]. In a later study, the clinical presentation of 78 individuals all with a loss-of-function mutation in ADNP was compared [45]. All patients had developmental delay, ID, mostly moderate to severe, and the greater majority of 93% had autism or autistic features. Individuals with *ADNP* disruptions were reported to have a less severe social affect symptoms compared to other monogenetic or idiopathic forms of autism. In the ADNP individuals, verbal intelligence explained to some extend the variance in social impairment [46, 47]. Behaviors in this group of 11 individuals with an ADNP mutations were characterized by high levels of stereotyped motor behaviors. Furthermore, within the ADNP group, age of walking predicted cognitive outcomes. A syndrome-specific sensory reactivity symptoms phenotype was identified, characterized by high levels of sensory seeking across tactile, auditory, and visual domains irrespective of age, sex, degree of autism, IQ, and adaptive behavior in a in an independent cohort of 22 affected individuals [48]. Additionally, using the Vineland Adaptive Behavior Scale, in a smaller cohort of 4 patients, implicated age-dependent developmental delays with increased impact of activities of daily living coupled to possible early neurodegeneration [46, 47]. Dysmorphic features included but were not limited to a prominent forehead with high anterior hairline, wide and depressed nasal bridge, and short nose with full, upturned nasal tip. More than 50% of patients suffered also from feeding and gastrointestinal problems, from visual problems, showed abnormal behavior, suffered from sleep problems, had hand or foot abnormalities, had brain abnormalities including seizures, had musculoskeletal issues such as scoliosis, joint laxity, and hip dysplasia, and was highly sensitive to infections of any kind [45; Table 1]. Apart from such frequent condition, a large minority of individuals with an ADNP mutations also suffered from congenital heart disease, ear–nose–throat system dysfunction, short stature, and abnormalities of the endocrine system. Of note, primary tooth eruption has been reported as accelerated [40]. The Helsmoortel–Van der Aa syndrome (OMIM # 615,873) can thus best be summarized as a complex neurological disorder that affects a multitude of organs and tissues [45, 49]. A permanently updated version of the clinical symptoms of the disorder can be found at the human disease genes website (https://humandiseasegenes.nl/adnp/). [50]

**Table 1:** Clinical features of individuals with a mutation in the ADNP gene

Intellectual Disability	100.0%
Speech delay	99%
Motor delay	96%
Autism Spectrum Disorder including autistic features	93%
Feeding and gastrointestinal problems^1^	83%
Behavioral problems	78%
Visual problems^2^	74%
Sleep problems	65%
Hand and foot abnormalities^3^	62%
Brain abnormalities including seizures	62%
Musculoskeletal system^4^	55%
Frequent infections	51%
Attention Deficit and Hyperactivity Disorder	44%
Congenital heart disease^5^	38%
Ear–nose–throat system^6^	32%
Urogenital problems^7^	28%
Short stature	23%
Endocrine system^8^	25%

^1^Including gastric tube feeding, oral movement problems, problems swallowing liquids, aspiration difficulties, lack of satiation, frequent vomiting, gastroesophageal reflux disease, constipation, and obesity. ^2^Including strabismus, nystagmus, ptosis, hypermetropia, myopia, cerebral visual impairment, and colobomata. ^3^Including nail anomalies, sandal gap, broad halluces, 2–3 toe syndactyly, brachydactyly, single palmar crease, prominent distal phalanges, prominent interphalangeal joints, polydactyly, interdigital webbing, 2–3 toe syndactyly, 5th finger clinodactyly, small fifth finger or absent distal phalanx of fifth finger, tapering fingers, broad fingers, fetal fingertip pads. ^4^Including scoliosis, joint laxity, hip dysplasia, Perthes disease, hip dislocation, pectus excavatum or carinatum and abnormal skull shape such as plagio-, trigono-, or brachycephaly. ^5^Including atrial septal defect, mitral valve prolapse, ventricular septal defect, patent foramen ovale, patent ductus arteriosus, tetralogy of Fallot, and unspecified cardiac defects. ^6^including narrow ear canals, hearing loss, frequent ear infections, ventilation tubes (grommets), adenoidectomy, tonsillectomy, and sleep apnea. ^7^Including renal anomalies, small genitalia, and cryptorchidism. 8Including signs of early puberty, growth hormone deficiency, and thyroid hormone problems. This table is based on data reported in our paper describing the clinical manifestation of the Helsmoortel–Van der Aa syndrome [45]

So far, only loss-of-function mutations such as stop-gain or frameshift mutations have been reported as unambiguously causative. Most, but not all mutations might give rise to a truncated protein [45, 51]. A single individual with a deletion of one copy of the entire ADNP gene has been described and the clinical presentation of this individual overlaps with those of loss-of-function mutations. The latter suggests that the Helsmoortel–Van der Aa syndrome could be due to a loss-of-function mechanism. However, as many mutations cluster in the fifth and last exon and escape from NMD has been demonstrated, the majority of patients might still produce protein. Thought it needs to be mentioned that mutated protein has never been unambiguously demonstrated in patients, an additional gain of toxic function of the mutant protein, if present, could also be envisaged [31, 51, 52].

The majority of mutations are unique and only observed in a single individual, so far. However, a few mutations, such as the p.Tyr719*, p.Arg730*, and p.Asn832Lysfs*81, are recurrent. Interestingly, patients with a p.Tyr719* mutation started to walk independently at a significantly later age had a higher pain threshold [45].

## The role of mosaicism in patients

The broad diversity of clinical presentations in some, but not all ADNP-mutant individuals is yet unexplained. That ADNP mutations arise as de novo mutations, where neither parent appears to have the mutation in their blood, begs the question as to how and when these de novo mutations arose. De novo mutations can arise in the germline, the developing embryo, fetus, child, or postnatally, throughout aging [reviewed in 53]. De novo mutations may present mosaically, with different mutation loads in different tissues. Such variations may vary among ADNP-mutant individuals. There are several studies of inter-tissue mosaicism of de novo mutations [54, 55]. A proper study of de novo mutations requires both access to multiple tissues and a sensitive quantitative means of mutation loads assessment [54]. Variable levels of mutation loads between affected and non-affected organs/tissues might explain the heterogeneous clinical spectrum of ADNP-affected individuals, as well as many de novo mutated ASDs.

## Opposing epigenetic signatures of the Helsmoortel–Van der Aa syndrome

Over the last years, methylation signatures specific for a series of neurodevelopmental disorders caused by mutations in a diverse series of genes have been identified [56]. When analyzing individuals with an *ADNP* mutation, uniquely two different and in part opposite methylation patterns were observed, depending on the location of the mutation in the gene. Mutations in the first half of the gene and mutations near the C-terminus of the protein result in a general pattern of approximately 6000 hypomethylated CpGs, whereas mutations in the central region of the gene resulted in a more limited set of approximately 1000 hypermethylated CpGs [57, 58]. It should be noted that no mutations in the intermediate region of the gene between aa 430 and 719 have been analyzed. Such methylation patterns established in patients with mutations in *ADNP* and showing confirmed symptoms of the Helsmoortel–Van der Aa syndrome have been postulated to be of value in determining the causality of apparent missense variants of unclear medical significance [59]. Of the six variants analyzed, only one de novo variant c.201G > C affecting the last nucleotide of *ADNP* exon 4 and predicted to affect splicing showed a methylation pattern consistent with the epimutations caused by loss-of-function mutations in the first half of the gene, suggesting that some missense mutation may not result in Helsmoortel–Van der Aa syndrome [57]. Unexpectedly, transcriptome analysis in blood cells did not identify clear differences between the two types of episignatures and a clear genotype–phenotype correlation between patients of either episignature was not identified [57, 58]. Interestingly, distinct phenotypes were associated with two mutations reflecting the different episignatures in cell culture [31], in line of a more severe phenotype observed in patients with the p.Tyr719* mutation (Box 1).

**Box 1:** Western blot detection of ADNP

Over the years, **ADNP detection** has remained far from unambiguous and different antibodies against the protein have been raised. Initially, ADNP was discovered as a novel Activity-Dependent Neurotrophic Factor (ADNF9/14)-like protein with a neuroprotective capacity exceeding that of ADNF9 itself. Here, ADNP was visualized on Western blot after incubation with an antibody raised against ADNF-14 (SALLRSIPA) [9, 60]. Based on its amino acid sequence, the theoretical molecular weight of ADNP without posttranslational modifications is estimated at 124 kDa (https://www.uniprot.org/). However, this SALLRSIPA antibody detecting ADNF-14 together with the NAP sequence resulted in a specific band signal of only 90 kDa at a Western blot. A molecular weight of around 90 kDa as observed on Western blot thus suggests proteolytic processing of ADNP. In 2001, a specific antibody raised against the synthetic peptide based on the ADNP sequence 989 to 1015 (CEMKPGTWSDESSQSEDARSSKPAAKK) resulted in a single band signal at 114 kDa, still well below the predicted molecular mass of ADNP [14]. Later studies reported visualization of ADNP Western blots with variable molecular weights ranging from 114 to 150 kDa, stressing the urgent need for more standardized and reproducible ADNP detection methods [13, 12, 30, 33, 41, 61, 62]

## ADNP interacts with multiple chromatin remodeling proteins complexes

**Fig. 3:**
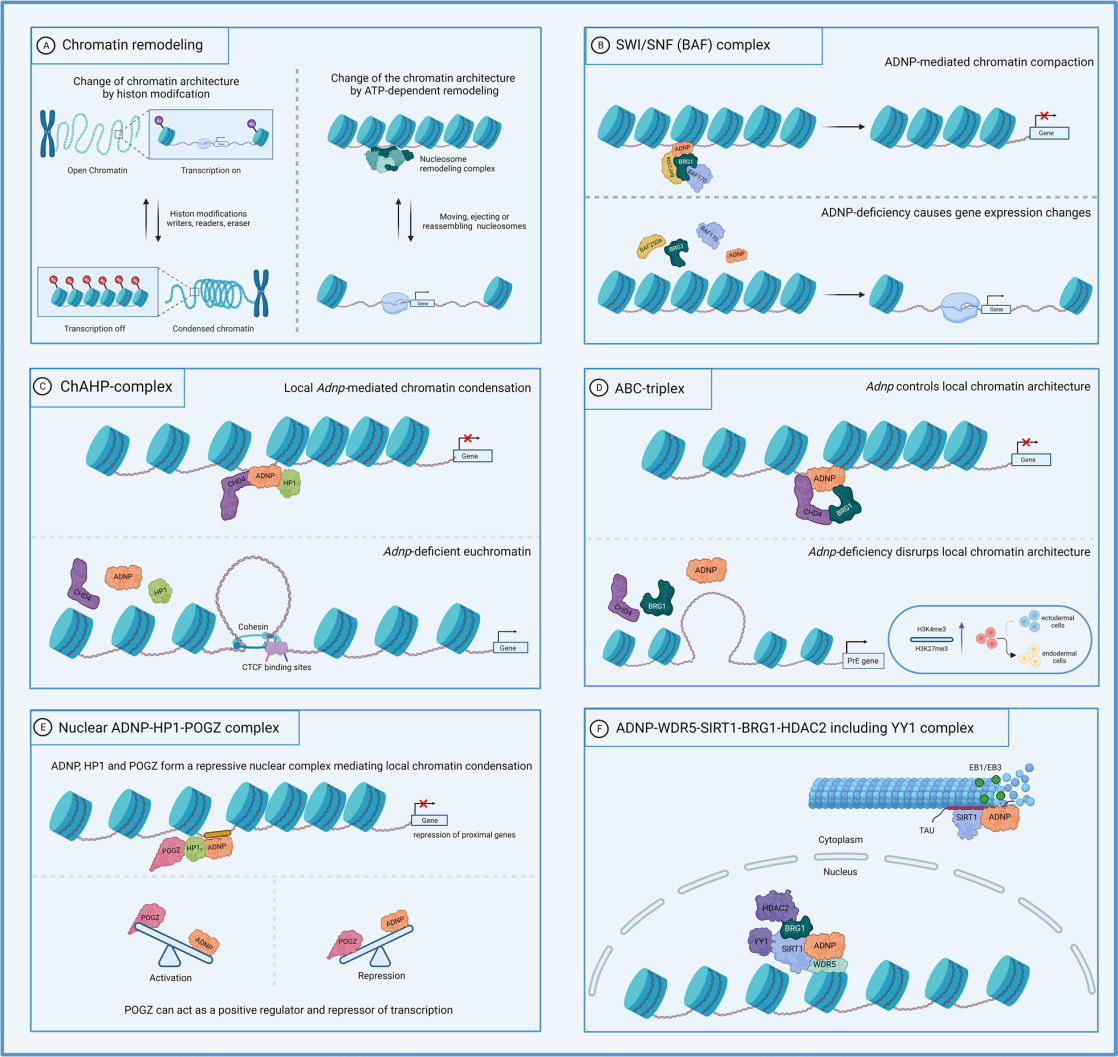
Overview of ADNP interactions with different chromatin remodelers.** (A)** General outline of chromatin remodeling, a dynamic process where changes in chromatin architecture can be modified by histone-enzymes such as writers, readers, and erasers. Change in chromatin conformation impacts the transcription machinery, e.g., open chromatin (euchromatin) is associated with gene transcription, while condensed chromatin (heterochromatin) is characterized by repression of transcription. Structural chromatin changes can be inducted by different chromatin remodeling complexes in an ATP-dependent manner, although non-ATP-related alterations have been identified (Box 2). **(B)** ADNP interacts with the ATP-dependent SWI/SNF (BAF) complex by its C-terminal portion with BRG1, BAF170, and BAF250a, thereby causing aberrant gene expression. **(C)** ADNP interacts with chromatin remodeler CHD4 by its N-terminus and HP1 by its C-terminus, forming a stable complex called the ChAHP complex, which masks local CTCF-motifs. Locally, the ChAHP complex mediates chromatin condensation at euchromatic regions, repressing stem cell differentiation-related genes. Complete *Adnp* deficiency results in disruption of the ChAHP complex and exposes the masked CTCF-motifs, normally bound by ChAHP, to introduce novel cohesion-insulated regions, resulting in chromatin recondensation, gene transcription, and spontaneous cell differentiation. **(D)** ADNP forms a stable triplex with BRG1 and CHD4 (ABC) which strongly binds to inaccessible chromatin. Loss of ADNP increases the ratio H3K4me3/H3K27me3 at key primitive endoderm (PrE) gene promoters, promoting differentiation toward endodermal cells. **(E)** The ADNP-HP1-POGZ complex is a nuclear repressive complex mediating local chromatin condensation. POGZ had a dual role as activator and repressor of transcription. High levels of POGZ and reduced levels of ADNP cause gene activation, observed as downregulation of proximal genes in *Pogz* knockout mice, while low POGZ levels and high ADNP levels cause gene repression, shown as upregulation of proximal genes in *Pogz* knockout mice. **(F)** In the nucleus, shared promotor region motifs were identified in the *ADNP* gene with *YY1*, *BRG1 (SMARCA4)*, and *HDAC2*, with *HDAC2* showing the highest similarity. Recently, WD repeat-containing protein 5 (WDR5) sites were found to be common between ADNP and chromatin modifier SIRT, mediating a nuclear-cytoplasmatic crosstalk and associating with microtubules/Tau

Chromatin structure is determined by a dynamic interplay between the DNA and cellular proteins. It can be modified by various mechanisms, including ATP-dependent chromatin remodeling (see Box 2) (**Fig. ****3****A**). Multiple studies have revealed an interaction between ADNP and members of different chromatin remodeling complexes. Accordingly, *ADNP* depletion leads to changes in chromatin structure and epigenetic variation in gene expression [39, 63–65]. However, the exact nature of the interactions between ADNP and proteins of different types of chromatin remodeling complexes remains ambiguous and five partially complementary theories have been put forward (**Fig. ****3**).

**Box 2:** Chromatin remodeler complexes

**Chromatin remodeling** controls access of various transcription factors and other relevant proteins to our DNA and has a crucial regulatory function enabled and maintained by at least for subfamilies of helicases: the switch/sucrose non-fermentable (SWI/SNF), the chromodomain helicase DNA-binding (CHD) family, the imitation switch (ISWI), and the inositol requiring 80 (INO80 complex) [66, 67]. All act by organizing and editing nucleosomes through ATP hydrolysis, rendering the DNA more or less accessible for transcription factors. In humans, and higher eukaryotes in general, the composition of each of these complexes is dynamic and may differ slightly depending on the cell type and developmental stage. During neural development, the SWI/SNF complex and the CHD family of chromatin remodelers are most active
The **CHD family** contains nine proteins, CHD1-9, each presenting with chromodomains. The family is divided in three subgroups. The first group, CHD1 and 2, has a C-terminal DNA-binding domain. The second group, CHD3 and 4, has N-terminal zinc fingers, and the last group, CHD 5–9, contains several domains including a DNA-binding domain, CR-domains, and SANT domains. This family of proteins can be part of a larger complex but can also act as a remodeler independently of other proteins. The proteins in this family are, for instance, involved in neural crest cell migration and synapse formation
The **SWI/SNF complex** (referred to as the **BAF complex** in mammals) contains 15 subunits with the core ATPase subunit consisting of either BRG1 (SMARCA4) or BRM (SMARCA2). The BAF complex can both inhibit and activate gene transcription, thereby playing an important role in the development of different tissues, especially in the neural system. The complex has been found to be important for neural progenitor proliferation, dendritic outgrowth, and axonal development
The **ISWI family** can form multiple small remodeler complexes. The ATPase subunit can be SMARCA1 or SMARCA5, of which SMARCA5 is the most abundant. The ISWI family is, like the other remodelers, important for nucleosome positioning and thus for regulating transcription and generate higher-order chromatin. Additionally, this family was also found to be important in the DNA damage response and thus for DNA repair [68, 69] The last family of chromatin remodelers is the **INO80 complex**, containing 15 subunits, forming three different modules. Only two of these modules are necessary for INO80 to perform nucleosome remodeling. They contain the two domains, Snf2-like ATPase/helicase and helicase-SANT-associated/Post-HAS, essential for ATP-dependent nucleosome remodeling [70]. Besides nucleosome remodeling and thus regulating transcription, the INO80 complex is also important for DNA repair and replication and exchanging histones [71]

### Interaction with SWI/SNF complex

Mandel and Gozes [12] demonstrated an interaction between ADNP and different proteins of the BAF chromatin remodeling complex. Human kidney HEK293T cells were transfected with recombinant ADNP fused to GFP at the N-terminus. Immunoprecipitation was performed with nuclear extracts using both anti-GFP and C-terminal ADNP antibodies which resulted in co-purification of ADNP among other binding proteins. However, C-terminal ANDP immunoprecipitation did not show binding partners, suggesting that the C-terminal part of ADNP is required for protein interaction. After size separation on polyacrylamide gels, additional protein bands were observed together with the 175 kDa GFP-ADNP fusion product. Mass spectrometry identified the BRG1, BAF250a, and BAF170 as co-precipitating with ADNP. The presence of these three subunits of the BAF complex, encoded by the *SMARCA4*, *ARID1A*, and *SMARCC2* genes, respectively, was confirmed by Western blotting. Multiple co-immunoprecipitation experiments were performed, with antibodies against a recombinant ADNP, fused to a GFP-tag, and against endogenous BRG1, all confirming that ADNP interacted with BRG1, BAF250a, and BAF170. In cellular stainings, co-localization of ADNP and BRG1 was seen, implicating a nuclear anchoring of ADNP to the SWI/SNF chromatin remodeling complex (**Fig. ****3****B**).

### ADNP is part of the ChAHP complex

Interaction of ADNP with the chromatin remodeler CHD4 and HP1 in a complex referred to as ChAHP was observed in ADNP-flagged mouse ESCs [39]. Binding with HP1 was previously reported during co-immunoprecipitation [63] and ChIP-seq experiments [72]. The study of Ostapcuk [39] showed that HP1*γ* was the most abundant isoform in the ChAHP complex, followed by HP1*β*. Interestingly, binding of the HP1*α* isoform was almost absent, whereas the study by Mandel [63] reported binding predominantly to HP1*α*. ADNP interacts with CHD4 through its N-terminus and with HP1*β*/*γ* through its C-terminus [39]. For this reason, it was speculated that HVDAS patients, who have C-terminal *ADNP* truncating mutations, could not form the ChAHP complex. Indeed, introduction of the most prevalent HVDAS mutation p.Tyr718* in the murine cells, mirroring the human p.Tyr719* mutation, retained binding to CHD4 but lost its ability to bind HP1. Since HP1 binds to H3K9me3, a silencing epigenetic mark common to pericentromeric regions, the HP1 might function to recruit its partners in the ChAHP complex to these repressive modifications. These findings confirmed earlier observations that ADNP binds the HP1-specific H3K9me3 histone mark in HeLa cells [21]. In the latter study, all three HP1 isoforms could act as a partner of ADNP in vitro but binding of ADNP could only be confirmed in mouse embryonic fibroblasts with the HP1*α* and *β* isoforms, suggesting the ADNP-HP1 interactions are influenced by additional cellular factors. Similarly, Ostapcuk found a ChAHP protein complex association between ADNP and CHD4 with H3K9me3-marked chromatin in mESCs. The ChAHP complex could still bind to its target genes when the chromodomain of HP1*γ* was mutated and binding of HP1*γ* to H3K9me3 was not altered in the absence of ADNP [39]. This suggested that ADNP recruits the ChAHP complex to specific sites in a sequence-specific manner. However, ADNP requires HP1 to repress gene transcription, which was shown upon generation of triple knockout cells for the three HP1 isoforms. These cells showed upregulation of specific genes that were also upregulated in the absence of ADNP only. These results are in line with ATAC-seq experiments revealing lack of ATAC-seq signals at ChAHP bound loci, suggesting that ChAHP is bound to non-accessible chromatin. In the absence of ADNP, these sites became accessible. Recently, an overlap in the peak spectrum of ADNP and CTCF sites was identified, indicating ChAHP-binding sites [65]. The absence of ADNP increased the CTCF signal, suggesting that ChAHP and CTCF compete for the same genomic binding sites (see below). By competition for CTCF sites, ChAHP reduces the accessibility of chromatin to prevent endodermal gene transcription in mESCs during neuroectodermal differentiation to ensure correct lineage specification. In the absence of ADNP, the ChAHP-binding sites became more accessible with neuronal progenitors expressing mesodermal markers instead of neuronal markers (**Fig. ****3****C**).

### ADNP-BRG1-CHD4 (ABC) triplex

More recently and in part contrasting with earlier reports, ADNP was reported to bind both CHD4 by its N-terminus and BRG1 by its C-terminus in mESCs [64]. This interaction was found by performing immunoprecipitation of ADNP followed by mass spectrometry using N-terminal and C-terminal ADNP antibodies. Subsequent ChIP-seq analysis of ADNP compared to ChIP-seq data for BRG1 and CHD4 showed that all three proteins were localized in similar genomic regions, including promotor regions and intergenic regions. In total, 31% of peaks overlapped between the 3 proteins. In concordance with earlier work, ATAC-seq experiments showed that ADNP bound to inaccessible chromatin. Consequently, loss of ADNP showed more ATAC-seq signals, indicative for accessibility to chromatin. However, these signals where not only linked to ADNP-bound loci. Therefore, these results rather suggest that ADNP is able to control chromatin architecture through its interactions with other chromatin remodelers. Previous studies confirmed contribution of ADNP-interacting chromatin remodelers BRG1 and CHD4 to bivalent histone modifications [73, 74]. Alterations of bivalent histone modifications in developmental genes in mESCs have been discovered in ChIP-seq experiments in the H3K4me3 and H3K27me3 markers [64]. Loss of ADNP increased the ratio of these histone marks, with a significant increase in the expression of the H3K4me3 marker over the repressive H3K27me3 mark, thereby forcing transcription at key primitive endoderm (PrE) gene promotors of, for example, *Gata6* and Sox7, resulting in prominent upregulation these genes and priming ESC differentiation toward endodermal cell types (**Fig. ****3****D**).

### The nuclear ADNP-HP1-POGZ complex

The autism/ID-risk gene *POGZ*, in which pathogenic variants have been associated with the White–Sutton syndrome, has been reported to interact with HP1 [75]. By co-immunoprecipitation of nuclear extracts of the embryonic mouse cortex, POGZ was shown to bind with HP1*γ*, but not with HP1*α*. HP1*γ* was also found to bind ADNP and CHD4 to form the ChAHP complex, which suppressed gene transcription [39 see above]. Using the Cut&Run technique, occupation of mutual chromosomal loci of *Pogz*, *HP1γ*, and *Adnp* was detected in the mouse embryonic telencephalon [76]. Thousand consensus loci were found in common among these three proteins, and overall these were associated with euchromatin as indicated by an enrichment for H3K27ac, but not for H3K9me3 characteristic of heterochromatin. Next, the presence of *Adnp* was investigated at loci where *Pogz* promotes expression of neuronal genes. Interestingly, reduced co-occupation of both *Adnp* and *HP1γ* with *Pogz* was observed at loci near genes that were downregulated in homozygous *Pogz* knockout mice. The proposed mechanism of the ADNP-HP1-POGZ complex involves *Pogz* promoting transcription at sites with less *Adnp/HP1y* co-occupancy but acting as a transcriptional repressor at sites with high ADNP/HP1y co-occupancy. The dual role of *Pogz* reflected on gene expression in *Pogz* homozygous knockout mice, where high ADNP-HP1-POGZ levels caused an upregulation of proximal genes, whereas high POGZ levels together with reduced ADNP/HP1y levels caused gene downregulation (**Fig. ****3****E**).

### ADNP-WDR5-SIRT1-BRG1-HDAC2 including YY1 complex

Most recently, several interactions sites of ADNP with SIRT1 were predicted, namely common interaction with WD repeat-containing protein 5 (WDR5), which mediates the assembly of histone modification complexes, as well as common interaction with BRG1 (see also above) and co-regulation by YY1 [22]. With common interaction partners and targeting of similar pathways such as TP53 and autophagy, the predicted ADNP-SIRT1 interaction was confirmed by immunocytochemistry in HEK293T cells and human iPSC-derived neurons. Moreover, immunoprecipitation experiments with EB1/EB3 antibodies in human neuroblastoma cells revealed co-elution of ADNP and SIRT1. However, in neuronal progenitor cells the EB1/3-SIRT1 interaction was initially lost but could be restored at low detection levels by administration of NAP, enhancing microtubule dynamics. Additionally, co-elution of Alzheimer protein Tau could be observed after EB1/EB3 immunoprecipitation, suggestive for a functional interaction of ADNP, SIRT1, and Tau, again enhanced after NAP administration. Mechanistically, exploring the promoter regions identified shared motifs among ADNP, YY1, BRG1 (SMARCA4), and HDAC2, with HDAC2 showing the highest similarity to ADNP (**Fig. ****3****F**).

## ADNP regulates microtubule functions

Microtubules (MT) consist of long cytoskeletal cylinders of polymerized tubulin, which are important for axonal plasticity and brain development [77, 78, 79]. An association between ADNP and microtubules has been reported in multiple studies [e.g., 80, 77]. End-binding proteins (EB) interact with MT-binding proteins and are important for axonal outgrowth, axonal transport, and dendrite formation. There are three EB family members (EB1, EB2, and EB3) of which EB3 is most abundant in the brain but both EB1 and EB3 are expressed in neurons. The latter two proteins are important for microtubule growth and interact with the SxIP motif (SIP, Ser-Ile-Pro), present in proteins such as ADNP to bind to MT and affect MT dynamics [19, 81].

Tau, a MT-associated protein important for tubulin assembly and MT stabilization, binds EB3 and regulates the localization and function of EB1 and EB3 in neuronal cells [82–85]. Tau is broadly expressed in neurons and is associated with different neurodegenerative diseases, collectively called tauopathies, like Alzheimer disease [83]. Interestingly, somatic ADNP mutations are directly correlated with increased tauopathy in the AD brain [85] and tauopathy was discovered in postmortem HVDAS child brain [86] as well as in mouse models with either *Adnp* haploinsufficiency [13] or carrying the p.Tyr718* mutation (the human p.Tyr719* orthologue [52]). In cell model systems, MT growth track length, and growth track speed of EB3 in cells that expressed the ADNP mutations, was decreased in speed and track length of EB3 compared to expression of full-length ADNP. Interaction of Tau with MTs was attenuated in the mutated cell lines [24, 86].

Other links between ADNP and Tau include increased mRNA and protein levels in the tauopathy mouse model compared to the wild-type littermates until the age of three months [61]. At 5.5 months, the ADNP level decreased. Tau has multiple isoforms, like 3R and 4R, which have, respectively, three and four repeats of the tubulin-binding motif. The ratio between these two isoforms is 1:1 in normal human brain. (And in normal mature mouse brain, the 4R tau is enriched.) The 1:1 ratio is disturbed in frontotemporal dementia, as was also seen in the tauopathy mouse model used above. These mice had increased levels of 3R mRNA at the age of three months, which disappeared in 5.5-month-old mice, paralleling the decrease in *ADNP* mRNA levels. The transgenic overexpression of mutated 4R in the mouse model is induced by the tetracycline-operon-responsive element and can be suppressed by doxycycline. After treatment with doxycycline, both *Tau* 3R mRNA and *ADNP* mRNA and protein levels normalized at the age of three months. Parallel experiments suggested an involvement of *ADNP* with *MAPT* (encoding Tau) alternative splicing with ADNP interacting with BRM and polypyrimidine tract-binding protein (PTB)-associated splicing factor (PSF)-binding, with PSF being a direct regulator of *Tau* transcript splicing [61].

In an independent study, increased *ADNP* mRNA levels were observed in certain brain regions in a six-month-old AD mice model [87]. The brain regions with increased ADNP levels were the regions where also A*β* plaques accumulated, possibly driven by A*β* toxicity. However, the option that these abnormalities were driven by Tau pathology was not excluded. Seemingly, in line with these observations, there is an increase of *ADNP* mRNA in lymphocytes in AD patients, possibly as a compensatory mechanism. Furthermore, higher premorbid intelligence was associated with greater serum ADNP levels [88]. In agreement with the latter observation and in a search for potential biomarkers, ADNP was the only protein reported as downregulated in the serum of patients with Alzheimer’s disease [89].

## Autism-mutated ADNP plays a pivotal role in autophagy by affecting cytoskeleton dynamics

Autophagy is a catabolic process via which superfluous or damaged proteins and organelles are delivered to the lysosome and degraded to release free amino acids into the cytoplasm. It is a highly conserved process, preserving cellular homeostasis by sequestering macromolecules and organelles into an autophagosome for delivery to a lysosome for degradation [90]. Subsequently, the degraded cargo containing nucleotides, peptides, and free fatty acids are released into the cytosol for metabolic reuse [91]. Defects in the autophagy pathway are associated in the pathophysiology of cancer, metabolic disorders, neurodevelopmental disorders as well as neurodegenerative diseases [92–95], and schizophrenia [23]. Several autophagy-related genes and proteins regulate the autophagy pathway, including Beclin 1 (BECN1), microtubule-associated protein 1 light chain 3 (LC3), and sequestosome 1/p62 (SQSTM1) [96]. BECN1 is responsible for autophagosome formation, while LC3 ensures maturation and closure of the autophagosome membrane. Moreover, LC3 is cleaved into LC3-I and the phosphatidylethanolamine conjugated LC3-II, which can be used as markers for the autophagy process. SQSTM1 ensures recognition of targets that are prone to autophagy by binding to ubiquitinated proteins, linking those to LC3 in the phagophore membrane [97]. In mice, ADNP has been shown to co-immunoprecipitate with LC3B after incubation with an ADNP antibody. The LC3B–ANDP interaction was partially lost in co-immunoprecipitation with brain extracts from *Adnp*^±^ mice [23]. Furthermore, *Adnp* haploinsufficiency reduced the expression of *Becn1* in the hippocampus [23]. Remarkably, autophagy was reported to be sexually divergent in an *Adnp* haploinsufficient model. Whereas RNA sequencing revealed negligible expression levels of *Becn1* in wild-type and *Adnp* heterozygous males, immunohistochemical studies identified a remarkable decrease in the expression of *Becn1* in *Adnp* heterozygous females in comparison with female littermate controls. In addition, the expression of *Becn1* could be differentiated between male and female mice, with females expressing a twofold higher hippocampal *Becn1* cells compared to wild-type males [98–100]. Similarly, a postmortem brain study in a 7-year-old male patient carrying a de novo mutation in the *ADNP* gene (described above has presenting tauopathy) revealed a decreased expression of *BECN1* in the cerebellum, mimicking the *Becn1* downregulation in *Adnp* haploinsufficient mice [86]. Finally, in a novel mouse model of *Adnp*, carrying the p.Tyr718* point mutation mimicking the most common human ADNP mutation, p.Tyr719*, FOXO3 was discovered as a new target of ADNP specific gene expression regulation with both ADNP and FOXO3 interacting with LC3 interaction [52] as well as with SIRT1 [22]. These findings suggest an association of autophagy, the cytoskeleton, and autism with regard to interaction with ADNP.

## ADNP and its regulating neuropeptide pituitary adenylate cyclase-activating polypeptide (PACAP) protect neurons against stress

Oxidative stress is caused by an imbalance in reactive oxygen–nitrogen species (ROS/RNS) overproduction and detoxification. Excessive oxidative stress plays a major role in brain aging and other unfortunate conditions such as hypoxia. The production of these radical molecules is detrimental for cellular structures, since they are potent of chemical modifications of nucleic acids, lipids and proteins, thereby causing neuronal cell death [101]. Oxidative stress models induced with peroxide showed an increase pro-apoptotic protein p53. Treatment with human recombinant ADNP protected neurons against the induced oxidative stress in conjunction with reducing p53 levels, thereby correlating fluctuations in ADNP expression to cellular protection [102]. Similar to ADNP, the expression of sister paralogue *ADNP2* also changed after peroxide administration in P19 embryonic cells. Silencing RNA-mediated knockdown of *ADNP2* resulted in reduced oxidative stress associated cell viability, indicating that *ADNP2* could play an essential role in cell survival pathways [33]. In addition, resistance to cell death was studied in malignant peripheral nerve sheath tumor (MPNST) cells, a lethal feature of patients suffering from neurofibromatosis type 1. Here, the neuropeptide pituitary adenylate cyclase-activating polypeptide (PACAP) induced an upregulation of ADNP, associated with a decrease in p53 expression [103]. It should be noted that PACAP and VIP (cited above as an ADNP regulator) share sequence and functional homology, and both have been shown to regulate ADNP [104]. Furthermore, the original cloning of ADNP associated it with p53 regulation and with specific amplification and expression changes in cancer [14]. Thus, it was suggested that PACAP/ADNP signaling is involved in tumor cell survival through p53 modulation. In nutrient-rich cultures, administration of exogenous PACAP38 induced an increase in *ADNP* expression in MPNST cells, which was shown to be associated with enhanced resistance to peroxide-induced cell death, reduced p53 and caspase-3 expression, together with less DNA fragmentation. Moreover, deprivation of nutrients enhanced resistance to oxidative stress which could not be ameliorated upon PACAP38 administration. These results indicate that nutrition deficiency forces MPNST cells to adapt to cellular stress by activating PACAP-driven ADNP signaling [103]. In an independent study, PACAP was discovered to regulate the stress-signaling axis in a sex-dependent manner. For this study, *Adnp* wild-type and *Adnp* heterozygous mice were treated with PACAP38 and subjected to stressful situations. Male heterozygous *Adnp* mice were more responsive to stress than females in object and social recognition. However, heterozygous females were less adaptive to stress in the open field and elevated maze tests. Pre-treatment with PACAP38 normalized the stress-evoked behavior in *Adnp* deficient mice. Moreover, splenic *Adnp* expression and plasma cortisol levels were regulated in a sex- and genotype-dependent manner, both correlated with cognition in male mice and anxiety in both genotypes. These deficits could be corrected by PACAP treatment, positively correlating to plasma cortisol levels. These findings were further investigated in a cohort of young men, where ADNP expression was positively linked to stressful situations and high salivary cortisol levels. These findings suggest ADNP as a biomarker for stress response assessment [105].

## The NAP motif in ADNP

NAP (Asn-Ala-Pro-Val-Ser-Ile-Pro-Gln, single letter code, NAPVSIPQ also known as davunetide, AL-108 and CP201) was originally identified as the smallest, conserved neuroprotective motif of ADNP [9, 14, 25], including amino acid overlap with the previously discovered activity-dependent neurotrophic factor (ADNF) [106, 107].

### NAP and microtubules

NAP associates with tubulin, a subunit protein of MTs, which are important for axonal plasticity and brain development [74, 77, 78, 79]. However, no interaction or influence of NAP on polymerization and dynamics of microtubules has been described [108]. The NAP structural similarity to cell penetrating peptides [77], coupled to proven dynamin-mediated endocytosis [109], also allows its binding to intracellular microtubule end-binding (EB1/3) proteins through its SxIP motif (NAPVSIPQ) [81]. ADNP mutations occur significantly more in AD brain than in control brains, and the effect of the mutations on microtubules was studied by growth track length, and growth track speed of EB3. In cells expressing the ADNP mutants, a decrease in speed and track length of EB3 compared to expression of full-length ADNP was observed. Treatment with NAP restored the tested parameters, while interaction of Tau with MTs was attenuated in the mutated cell lines. Furthermore, the MT-NAP interaction is associated with protection against autophagic deficits, with ADNP binding to microtubule-associated protein 1 light chain 3 (LC3) augmented by NAP (described above) [23, 98, 100, 110, 111]. In this respect, NAP partly corrects RNA transcript expression levels that are deregulated as a consequence of ADNP deficiency/mutation including the autophagy and microbiome resilience-linked forkhead box 3 (FOXO3) [112]. This NAP-related transcript regulation may be linked to cell penetration and regulation microtubule dynamics, thereby influencing mRNA levels [109, 113]. The NAP SxIP motif allows further interaction with other SxIP motif-containing proteins, like ADNP [98, 114], and the FOXO3 partner sirtuin 1 (SIRT1) [115] to increase EB1/EB3 and Tau-microtubule binding [22], axonal transport, and dendritic spine formation [114, 116]. Dysregulations of SIRT1 [117], FOXO3 [118], and ADNP [22] are also related to human aging and neurodegeneration. Interestingly, NAP/ADNP regulate tubulin isotype expression levels [98, 112, 119], with increased microtubule microheterogeneity linked to neuronal/brain development [120, 121].

**Box 3:** Animal models

First attempts to study the *Adnp* gene function was initiation by generation of a complete **knockout mouse model** by incorporation of a neomycin cassette in exons 3–5 in a 129/Sv-derived embryonic stem cells, crossed with C57BL/6 mice and propagated on a mixed genetic background [11]. Full knockouts, lacking the entire protein coding sequence of the *Adnp* gene, are embryonically lethal and die around E8.5–9.0 with failure of the neural tube closure, suggesting a role in brain development. For this reason, **heterozygous male-mutant mice** were created which are **haploinsufficient** for one of the two *Adnp* alleles. These mice showed the expected 50% reduction in *Adnp* RNA and protein levels and exhibited cognitive deficits in the Morris water maze in adolescence and older age [13]. No obvious phenotypic abnormalities were mentioned [13], be it that in follow-up studies a small reduction in body size of the mutant animals was measured [122]. However, in the brain, an increase neuronal degeneration markers, some of which reminiscent of tauopathy, was noted. The heterozygous phenotype exhibited a distinctive pattern of abnormally regulated genes. Most notably, Affymetrix array-based expression profiling demonstrated upregulation of a gene family encoding for proteins enriched in the visceral endoderm such as apolipoproteins, cathepsins, and metallothioneins, and downregulation of organogenesis markers including neurogenesis and heart development [63]. When both sexes were analyzed in subsequent experiments, it is striking that male heterozygous knockout mice exhibited deficiencies in an object recognition and social memory test, whereas females were at least partially spared [11]. It should be noted here, though, that in all latter experiments due to a reduced reproductively of the *Adnp* colony in a mixed genetic background, breeding was continued in ICR outbred mice. Using manganese-enhanced magnetic resonance imaging, axonal transport abnormalities were reported in haploinsufficient mice of both sexes, but transport was reported faster in females than in males [98]. Furthermore, haploinsufficient mice showed reduced dendritic spine density, vocalization impediments, gait, and motor dysfunctions [122]
While it is tempting to draw parallels between the clinical presentation of patients and the abnormalities observed in the mouse model, it should be stressed that the model described is a full deletion and the mutational mechanism of the Helsmoortel–Van der Aa syndrome has not been fully established. For this reason, a mouse model mimicking the most frequent human p.Tyr719* mutation was generated. CRISPR/Cas technology introduced a stop codon at the equivalent position in the mouse genome [52]. The p.Tyr718* mutation is surrounded by a minimal non-coding sequence alterations to facilitate the genome editing process. **Heterozygous Tyr mice** in a congenic C57BL6/N background express the Tyr mutated allele and the wild-type allele at 50% reduction each on the mRNA level. A truncated protein product in the mutant animals was predicted but not visualized. Phenotypically, Tyr mice had delayed development and with sex-dependent gait defect and syntax abnormalities. Grooming duration and nociception threshold autistic traits were significantly affected in males. Anatomically, dendritic spine densities were reduced an morphologies altered. Early-onset tauopathy in hippocampus and visual cortex was accentuated in males and was paralleled by impaired visual evoked potentials. Expression profiling highlighted among many other dysregulated genes, the decrease in expression of the *Foxo3* gene, involved in autophagy
Most recently, we generated a humanized model for the Helsmoortel–Van der Aa syndrome by CRISP/Cas endonuclease mediated genome editing to delete a 14 nucleotide sequence introducing a frameshift mutation just distal to the NES. Mice carrying the heterozygous frameshift p.Leu822Hisfs*6 mutation are viable and fertile and initial investigations show it may become a valid model to study the human condition (D’Incal, Vanden Berghe and Kooy et al., *manuscript in preparation*). The model is available from the Jackson laboratories as stock 033,128

### The ADNP, NAP, and Tau interaction

Tau is a microtubule-associated protein important for tubulin assembly and MT stabilization, regulating the localization and function of EB1 and EB3 in neuronal cells [19, 83–85]. Tau is broadly expressed in neurons and is associated with different neurodegenerative diseases, called tauopathies, like Alzheimer’s disease [83]. By both binding to EB3, NAP influences Tau-MT association and can protect MTs [85, 123]. The correlation between NAP and Tau is further confirmed by multiple studies that show that NAP can protect against tauopathies. For example, Tau phosphorylation was increased in heterozygous *Adnp* mice in comparison with *Adnp* wild-type mice [13]. Similar increases, including increases in Tau deposition (tauopathy), were observed in the Tyr mice, mimicking the most abundant ADNP mutation in children [52]. After intranasal NAP treatment in the mouse models above (and below), as well as in an AD mouse model, a decrease in Tau hyperphosphorylation and a beneficial effect on A*β* aggregation in an early state of the disease (9–12 months) were observed [124, 125]. NAP also increased Tau-MT association in the case of zinc intoxication in cell cultures [123].

## Candidate drugs for the Helsmoortel–Van der Aa syndrome

NAP compensates for deficits linked with mutated human ADNP in cell cultures (e.g., MT deficits with truncated ADNP p.Tyr719*, p.Arg730*, and p.Ser404*) [86, 126, 127]. Correspondingly, NAP protects cellular survival and induces neurite outgrowth [119, 128] as well as dendritic spine formation [81, 112, 122], culminating in synaptogenesis [129], which is implicated in neurodevelopmental diseases and autism spectrum disorders (ASDs) [130]. Moreover, NAP, as well as the shorter NAP interacting derivative SxIP motif-containing SKIP (Ser-Lys-Ile-Pro) [98], is beneficial in the haploinsufficient *Adnp* mouse model, protecting behavior [98, 122], with NAP reducing Tau hyperphosphorylation in the two-month-old haploinsufficient *Adnp* male mouse brain [13]. This is paralleled by NAP/SKIP protection of MT-ADNP and sex-dependent axonal transport [98, 131], and with decreased testosterone levels paralleling increased tauopathy in humans [132]. In turn, tauopathy correlates with decreased cognition and ADNP levels [88], and increased somatic mutation loads in the aging/human Alzheimer’s disease brain [126].

The haploinsufficient *Adnp* mouse model (see Box 3) is now complemented by a novel genome edited (CRISPR/Cas9) Tyr-mouse harboring the most prevalent HVDAS mutation (p.Tyr719*, corresponding to mouse p.Tyr718*) [52]. The Tyr-mouse displays phenotypes attributable to heterozygous expression of 50% wild-type *Adnp* (loss of function) and 50% Tyr-*Adnp* (potential gain of toxic function) alleles. As indicated above, young (~ 2-month-old) Tyr mice displayed Tau deposition, which was coupled with visual evoked potential (VEP impairments) and amelioration by NAP treatment [52]. Vocalization/communication deficits in *Adnp* haploinsufficient and Tyr pups [122] were previously correlated with mouse aging/tauopathy [133], connecting aberrant synaptogenesis to synaptic loss and neurodegeneration. The Tyr-mouse findings recapitulate the human tauopathy in a seven-year-old-postmortem ADNP p.His559Glnfs*3 brain [86]. The demonstrated sexual dichotomy of *Adnp* haploinsufficient mice [23, 98, 105, 122] is further enhanced in the Tyr-mouse. Developmental delays and gait dysfunctions were increased in Tyr females compared to *Adnp*^±^ mice, with adult muscle knockdown of *Adnp* resulting in robust female gait defects, corrected by NAP [134], coupled with sex-muscle-specific differentially expressed genes, also partly corrected by NAP [135]. The mouse models suggest peripheral biomarkers, like FOXO3, and with FOXO3 regulating the microbiome, the ADNP/NAP peripheral biomarkers include a microbiota signature [136]. With extensive preclinical pharmacology, clean safety, pharmacokinetic profile for the intranasal and intravenous administration routes (half-life of 30–120 min) [137–139] coupled to clinical efficacy in amnestic mild cognitive impairment and schizophrenia patients [139], and FDA orphan drug and pediatric rare disease designations, the path is cleared for NAP clinical development for the HVDAS.

Other drug candidates developed for the HVDAS include ketamine (ClinicalTrials.gov Identifier: NCT04388774). The ketamine rationale is based on the finding that ketamine increased ADNP expression, however, the possibility of ketamine increasing mutated *ADNP* expression needs to be further investigated [140–142]. Meanwhile, an open label trial of a low doses of ketamine in ten individuals affected with the Helsmoortel–Van der Aa syndrome demonstrated that the drug is well tolerated and considered safe [143]. A nominal improvement in the Clinical Global Impressions—Improvement Scale, was reported, which according to the authors should be interpreted with care due to the study limitations of a small cohort size and the absence of a control group.

## ADNP dysregulation in cancer development

Many transcription factors and chromatin regulators that are hit by driver mutations in cancer are also targeted by de novo germline mutations that cause neurodevelopmental syndromes comprising systemic phenotypes along with intellectual disability (ID) and autism spectrum disorder (ASD) [144, 145]. Paradigmatic examples include chromatin regulators such as KMT2D [146], GTF2I [147], EZH2 [148, 149], YY1 [150, 151], and subunits of BAF complex [152, 153]. Similarly to high prevalence of mutations in genes encoding subunits of the SWI/SNF chromatin remodeling complexes or other epigenetic regulators in cancer development, increasing evidence also points to an oncogenic role for *ADNP* mutations [25, 154–156].

**Fig. 4:**
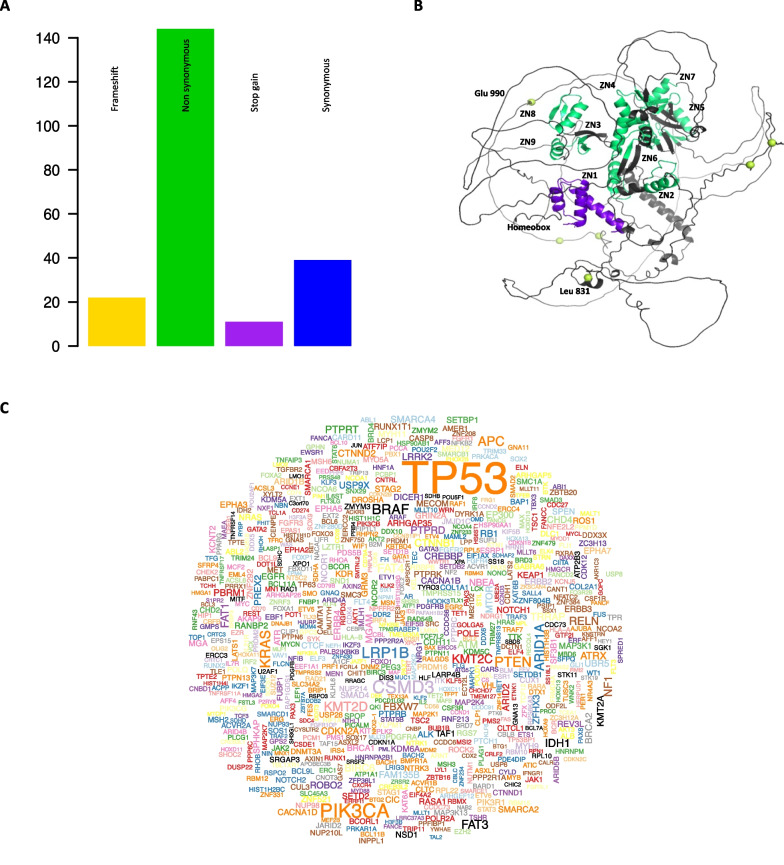
Cancer-related associations of ADNP. **(A)** Table of types of *ADNP* mutations found in PanCancer. **(B)** 3D structure of ADNP retrieved from Alphafold database; light green domains represent zinc (ZN) fingers and the purple domain represent the homeobox domain; lime yellow spheres represent alpha carbon of residues in which genetic mutations cause frameshifts and incorporation of a premature stop codon. Leu 831 is reported as a reference position of hot locations that are often mutated into early stop codon/frameshift causing HVDAS. **(C)** Word cloud adapted from the OncoVar website, referring to top cancer drivers, which includes several known ADNP interactors

In total, 216 ADNP mutations are displayed in the PanCancer database thanks to the OncoVar platform (https://oncovar.org/), which employs published bioinformatics algorithms and incorporated known driver events to identify driver mutations and driver genes (**Fig. ****4****A**) [157]. None of those has actually been defined as driver but the gene has been given a driver level score of 2 (i.e., likely pathogenic), and it has been independently identified as a tumor suppressor [142]. The gnomAD database reports mutations that cause loss of function (LoF) and annotate those that do not apparently lead to haploinsufficiency; for instance, mutations are predicted to escape from NMD as ‘not LoF’ [158]. Six frameshift mutations have been curated as not LoF because they end up in the last exon. ADNP has a DNA-binding activity and no catalytic function. Hence, the ablation of its external domains (i.e., likely responsible for protein–protein interaction) caused by these 6 ‘not LoF’ could indeed strongly impact protein function. Cancer-specific missense mutation A1866D in CHD4 C-terminus domain causes a reduction of CHD4 interaction with ADNP, suggesting that ChAHP formation might be perturbed in cancer cells [159]. In fact, CHD4 and several other ADNP interactors are among the top driver genes in the Cancer Genome Atlas (TCGA) (**Fig. ****4****C**).

On a structural point of view, cancer-causing ADNP mutations seem distributed all over the protein in the primary structure, but they are close to each other at the tertiary structure (**Fig. ****4****B**). NDD-causing mutations affecting protein structure are overrepresented near the ADNP homeobox domain (**Fig. ****4****B**), suggesting that both cancer and neurodevelopment entail the disruption of ADNP chromatin-binding activity. Still, we can observe that most ADNP cancer-causing mutations affect the protein primary structure, while several NDD-causing mutations are displaced all over the gene body, suggesting that the former might be more related to the selection of mutations that affect protein activity modulation, while the latter are more generally inactivating. However, it cannot be excluded that the distinct pathogenic outcomes of these mutations, in terms of neurodevelopment or cancer, might rather be a consequence of the time and space of emergence of these mutations during development, like it has been observed for other neurological conditions [160], affecting individuals early—in germinal or multipotent cells during embryogenesis—or late, in somatic cell, during adult life—respectively.

Overall, there is increasing consensus that ADNP contributes to tumorigenesis and cancer progression. Transcriptional assessment of *ADNP* levels in a large cohort of bladder cancer specimens highlighted a significant overexpression in the patients with tumor progression and the association of higher *ADNP* protein levels to poor prognosis in such patients [161]. In bladder cancer, ADNP promotes tumor cell growth and proliferation by enhancing the AKT-MDM2-p53 molecular axis which stabilizes cyclin D1 and ultimately leads to cell cycle acceleration from G1 phase to S phase [162]. In another study, ADNP was found to promote bladder cancer cell migration via TGF-*β*-mediated epithelial-mesenchymal transition (EMT) pathway [161]. In that respect, it is relevant to mention that ADNP was recently identified as a binding partner of the ZEB1/NuRD complex (zinc finger E-box-binding homeobox 1/Nucleosome Remodeling and Deacetylase) which is critically involved in epigenetic cancer programming of EMT hallmarks [163]. ADNP overexpression has also been linked to the pathogenesis of germinal center B cell-type diffuse large B cell lymphoma (GCB-DLBCL); genome-wide screening of cancer-associated super-enhancers in this cell type and other human B cell lymphoma lines revealed the upregulation of *ADNP*, which is significantly associated with increased risk of death. Moreover, *ADNP* knockdown in GCB subtypes of B cell lymphoma inhibited proliferation, overall suggesting that epigenetic super-enhancer-mediated control of ADNP contributes to GCB-DLBCL progression [164]. ADNP is also part of a protein hub that is enriched in the signal transduction of pathways involved in colorectal cancer (Rahman et al., 2019). In such context, silencing of *ADNP* primarily affected Wnt-signaling genes by increasing the expression of many of its effectors such as the tumor drivers DNMT1 and TALIN-1, thus showing an ADNP-mediated repression on the Wnt axis. Moreover, downregulation or depletion of *ADNP* both in vitro and in tumor xenografts showed increased migration, invasion and proliferation of colon cancer cells resulting in accelerated tumor growth, suggesting a tumor suppressor function of ADNP, and highlighting its potential role as a prognostic biomarker for colorectal cancer. Moreover, pharmacological administration of subnarcotic dosage of ketamine induces *ADNP* levels thus suppressing tumor growth [142, 165]. Another example of ADNP involvement in aberrant tumorigenic mechanisms is provided by the Malignant Peripheral Nerve Sheath Tumor (MPSNT). This is a highly aggressive comorbid manifestation associated with Neurofibromatosis type 1 and is characterized by abnormal growth under prohibitive metabolic conditions. In the lack of growth-stimulating factors typical of MPSNT niche, the higher endogenous level of PACAP induces the overexpression of ADNP, which in turn provides a protection against H_2_O_2_-induced oxidative stress, thus mediating an adaptive mechanism that contributes to tumor cell resistance [103]. Finally, genomic and proteomic analyses in High-Grade Serous Ovarian Cancer (HGSOC) cells identified *ADNP* as a novel altered transcription factor whose gain in DNA copy number and increase in mRNA and protein levels are associated with poor prognosis. *ADNP* expression is required for cell viability and strongly associated with tumor maturation by impacting on cell cycle progression and proliferation pathway. Indeed, silencing of *ADNP* markedly reduced cell growth and colony formation, along with a concomitant increase of apoptotic events. To further corroborate this hypothesis, functional enrichment of downregulated genes upon *ADNP* silencing includes fundamental modulators of cell cycle checkpoints such as CDC25A, a key regulator of both G1/S and G2/M transitions, whose protein levels are reduced upon *ADNP* silencing [166].

## ADNP chromatinopathies disturb higher-order 3D chromatin landscapes

The epigenetic mechanisms by which ADNP chromatinopathies contribute to pleiotropic clinical presentations of ASD, cancer, and neurodegeneration still remain poorly understood [5, 167]. Recent studies have shown that chromatin remodeling complexes play an important role in higher-order chromatin structure in neuronal development and that disruptions during development influence the maturation of neural networks [7, 168–171]. During cell differentiation, the 3D genome architecture changes dynamically and subsequently alters the regulation of gene expression [172–176]. At the genome level, euchromatic from heterochromatic regions are organized into chromosome territories. These megabase-sized chromatin domains are organized into smaller and smaller subdomains known as topologically associated domains (TADs) [173, 177]. Long-range chromatin contacts that bring genes and regulatory sequences in close proximity in TADs are necessary for co-transcription of biologically related and developmentally co-regulated ASD genes [64, 65, 178–181]. Recent advances in methodologies based on chromatin conformation capture (3C-Hi-C) start to provide new perspectives on the genome-wide 3D chromatin organization of ADNP chromatinopathies in pathological developmental processes [174].

For example, CTCF-binding sites are known to be spread across the genome by transposable elements (TEs), allowing the formation of complex 3D chromatin architecture via large domains of chromatin, organized into TADs [65, 173, 177]. CTCF in concert with cohesin regulates 3D chromatin loop organization in the genome [65]. Within TADs, long-range chromatin interactions can be created, bringing gene and regulatory sequences together which are required for the co-transcription of ASD genes. These interactions can be disrupted by mutations in chromatin remodelers such as ADNP, an active participant of the ChAHP chromatin remodeling complex. It has been assumed that ADNP, and the entire ChAHP complex, has an important evolutionary role as it prevents rewiring of 3D genome architecture, thereby allowing TE-mediated CTCF motif spreading. ATAC-seq showed that ADNP inhibits cohesin-mediated looping by competing with CTCF at TAD boundaries in *Adnp* knockout versus wild-type mouse ESCs. Furthermore, 4C-seq experiments have revealed that *Adnp* knockout causes loss of the *Caudal-type homeobox 2 (Cdx2)* gene which is involved in trophectoderm specification and maintenance. In wild-type mESCs, the *Cdx2* promotor is looped to a locus enriched for histone H3 lysine-27 (H3K27) acetylation, indicating that it is an active enhancer. These H3K27 acetylation levels remain unchanged following *Adnp* deletion, whereas CTCF and cohesin binding creates a strong loop, weakening the promotor–enhancer interaction, leading to a reduction in *Cdx2* expression. In line, *Adnp* knockout mouse embryos showed impaired formation of the neural tube, which leads to lethality around embryonic day 9.5 [11, 39]. In this respect, it could be argued that different phenotypes in humans with heterozygous *ADNP* mutations, as seen in HVDAS, could be a consequence of directly affected local perturbations in 3D chromatin organization [65]. Alternatively, ADNP has also been identified as an interaction partner of RNA polymerase III transcription factor C (TFIIIC), which recognizes acetylated Alu elements (AEs) that interact with CTCF-binding sites and show promotor–enhancer functions [182]. This has been investigated by comparing ATAC-seq data from T47D cancer cells growing in normal conditions with T47D cells after 16 h of serum starvation (SS) [182]. Upon SS, TFIIIC is recruited by AEs pre-marked by ADNP, which alters their chromatin accessibility by direct acetylation of H3K18 [182]. Therefore, ADNP together with CTCF could be involved in TFIIIC-mediated long distal looping, supporting the hypothesis that ADNP has a pleiotropic role in 3D chromatin shaping [182]. These interactions may give rise to obvious differences between primates and rodent models, with sequence specificity; however, it is interesting to remember that at the mRNA level *ADNP* is 90% identical between mouse and humans [14].

Another particular example of higher-order chromatin structure, contributing to genome instability, is the R-loop, defined as a three-stranded nucleic acid structure accumulating on chromatin. These arise as a consequence of transcription, and are comprised of a DNA/RNA hybrid together with a displaced single-stranded DNA (ssDNA) [183–186]. The displaced ssDNA can fold into a G-quadruplex structure, stabilizing the R-loop and playing a regulatory role during transcription and DNA repair [186]. Temporary regulation by R-loops is important in essential physiological processes such as regulation of gene expression, immunoglobulin class switch recombination, and mitochondrial replication initiation [187, 188]. Importantly, these R-loops should be preserved as they are essential for the regulation of biological relevant processes, while harmful R-loops, associated with several disorders as mentioned above, should rather be eliminated. Elimination of harmful R-loops is facilitated by several factors such as helicases which unwind the DNA/RNA hybrid or G4 structures and ribonuclease H (RNase H) enzymes which directly degrade the RNA within the DNA/RNA hybrids to form double-stranded DNA (dsDNA) [186]. Using a proximity-based labeling approach in vitro, ADNP was found to be highly enriched close to RNase H, proving it directly regulates R-loops. ADNP is involved in R-loop suppression with its associated zinc finger motifs resolving R-loops, while the homeodomain is directing ADNP to chromatin. Several mutations associated with Helsmoortel–Van der Aa syndrome result in loss of the homeodomain, but not of the zinc finger motifs [10, 45]. Without the presence of the DNA-binding homeodomain, ADNP is unable to localize to chromatin associated with R-loop deregulation. It has been found that R-loops accumulate at the ADNP target, which suggests haploinsufficiency is a main underlying cause of HVDAS [186]. However, gain of toxic function is a complementary, confounding phenotype, as described for the currently known, most abundant *ADNP* p.Tyr719* mutation (p.Tyr718* in mice) truncating the protein at the nuclear localization signal (NLS) and leading to a complex phenotypic outcomes [52].

## A concluding look ahead

In this review, we discussed the current knowledge of the chromatin remodeler *ADNP*, a gene frequently mutated in syndromic autism. Since its discovery, we demonstrate the multimodal aspect of ADNP in various processes by interaction with its protein domains, e.g., regulation of the cytoskeleton by the NAP motif, heterochromatin formation by its PxVxL interaction motif, and DNA binding by the presence of a homeobox domain and zinc fingers. Moreover, we described binding of ADNP to proteins involved in autophagy (LC3), autism pathways (EIF4E/SHANK3), chromatin remodeling (BRG1/HP1/CDH4), and epigenetic modifying genes (SIRT1/HDAC2/YY1), all impacting the physiological status of the cell. Of special note, chromatin remodeling complexes are key regulators of the unfolded protein stress autophagy response to promote clearance of cytoplasmic protein aggregates [189]. Maintenance of protein folding homeostasis, or proteostasis, is crucial for cell survival as well as for execution of cell type-specific biological processes such as neuronal synapse and memory formation, and cell transition from a mitotic to post-mitotic cell type. Besides, several epigenetic chromatin enzymes were recently shown also to remodel the cytoskeleton, regulating structure and function of microtubules and actin filaments [190]. This points to an emerging paradigm for dual-function remodelers with ‘chromatocytoskeletal’ activity that can integrate cytoplasmic and nuclear functions. This epi-chromato-cytoskeletal regulation suggests that cells coordinate epigenetic differentiation programs with acquisition of specialized cytoskeletal structures [191]. Such coordination could affect brain development by coordinating patterns of gene expression with remodeling of the neuronal cytoskeleton. This, in turn, could modify such basic neural functions as learning, memory, and behavior [190, 192].

The nuclear function of ADNP is most often ascribed to chromatin remodeling and shaping the 3D chromatin structure, and the protein has been identified in a multitude of epigenetic complexes. With confirmed interactions in the SWI/SNF and ChAHP complex, ABC-triplex, and POGZ, ADNP was independently associated with binding of HP1, remarkably, in each of the mentioned chromatin remodeling complexes. However, there are contradicting reports whether the *α*, *β*, or *γ* isoform of HP1 is the main interactor. Here, we postulate that which HP1 subunit ADNP binds is dependent of the tested sample material (e.g., cell lines or tissue), species (e.g., mouse or human), and the implemented technique (e.g., CoIP-MS, ChIP-seq). The remarkable interaction with POGZ, of which mutations are causative of the White–Sutton neurodevelopmental disorder, shows striking associations with molecular dysfunctions found in the Helsmoortel–Van der Aa syndrome. Therefore, we stress the necessity for studying the role of the nuclear ADNP-HP1-POGZ complex in the Helsmoortel–Van der Aa syndrome and how mutation of ADNP reflect on the molecular impact of POGZ. Lastly, computational analyses have revealed WRD5-binding sites in ADNP associating it with the epigenetic SIRT1 enzyme. Binding was confirmed by co-immunoprecipitation. Additionally, promotor motif alignments identified similarities of ADNP with YY1, BRG1, and HDAC2. With a confirmed binding to BRG1, chromatin remodelers YY1 and HDAC2 still lack evidence for direct binding to ADNP as well as the impact of HVDAS-related stop mutations on this predicted novel epigenetic complex.

Constitutional stop and frameshift mutations in *ADNP* cause a form of syndromic autism referred to as the Helsmoortel–Van der Aa syndrome. Most mutations are present in the last exon and escape from NMD. Although mutant mRNA has been detected in patient-derived sample materials and mouse models, we still lack evidence for the presence of a truncated ANDP protein in patient material, though ADNP-mutant protein has been detected in cellular systems when the protein has been engineered to include a Flag® or GFP-tag. The absence of detectable mutant protein could indicate a loss-of-function mechanism. This would be compatible with the relative uniform clinical presentation of individuals with the Helsmoortel–Van der Aa syndrome [45]. However, a presumed uniform mutational mechanism of the disorder is hard to combine with the partially opposing methylation signatures in blood of patients in function of the location of the mutation [57, 58] and potentially with a slightly more severe clinical presentation of patients with a p.Tyr719* mutation [45]. Here, patients with N-terminal mutations as well as with C-terminal mutations showed an overall hypomethylation pattern. However, mutations in the central part of the protein rather presented with a general pattern of hypermethylation. Interestingly, the mutation boundary between hypo- and hypermethylation near the C-terminus is coinciding with the NLS, with mutations before the NLS resulting in hypo and the mutations after the NLS resulting in hypermethylation. This boundary could theoretically be explained by a truncated mutant protein being generated and entering the nucleus if the NLS is present or remaining cytoplasmatic, when the NLS is absent. Overexpression of (mutant) GFP-tagged ADNP in cellular systems confirms that the presence of the intact NLS is responsible for entry in the nucleus. However, such a theoretical model, mutant protein has never been detected in patients, would point to an (additional) gain-of-function/dominant negative function of the *ADNP* mutations. However, this model cannot explain why N-terminal mutations have an epigenetic effect in patients similar to C-terminal mutations and the mutational mechanism remains to be unveiled.

The *ADNP* paralogue *ADNP2* has not (yet) been linked to any disorder, although some *ADNP2* variants were named associated with the onset of schizophrenia [17]. Currently, only one de novo 4.8 Mb heterozygous microdeletion has been identified on chromosome 18q23, taking away the *ADNP2* gene among others [193]. The individual with this deletion had a clinical presentation including congenital glaucoma, bilateral microphthalmia, pupil and iris anomalies, dysmorphic facial features, mild cognitive impairment (IQ about 60), congenital deafness. The patient also inherited a pathogenic frameshift mutation in *FOXC1* from the father. While the *FOXC1* mutation can explain the eye phenotype of the patient, the origin of the other clinical symptoms remains unknown at the moment, but a role for the *ADNP2* gene cannot be excluded.

The dual role of *ADNP* mutations in both neurodevelopment and cancer suggests that altering the core circuitry regulating differentiation has vastly divergent, developmental stage-dependent consequences, with equivalent mutations resulting, in developmental delay or in cancer, depending on whether they are present throughout development or arise after establishment of mature somatic lineages. A thorough meta-analysis of brains from ASD individuals revealed a common ground gene expression dysregulation and biological pathway derailments in cancer [194]. The opposite tendency of developing one condition or another (here ASD and cancer, respectively) within a population is called *inverse comorbidity*. Extending the case to central nervous systems (CNS) disorders in general [160], large observational studies showed that this is true for several common neurological disorders, and it might be relevant to assess in the case of ADNP. Oncogenic mutations can be exquisitely sensitive to developmental context, as most vividly demonstrated by nuclear transplantation experiments in mice in which egg-mediated reprogramming was able to suppress, through the blastocyst stage, the oncogenic potential of mutant genomes from leukemia, lymphoma, and breast cancer cells [195]. These findings suggest that the regulatory circuitry operating in pluripotent stem cells could counteract the effects of oncogenic mutations, introducing some sort of epigenetic robustness toward cancer that in turn affects neurodevelopment. The possibility to obtain iPSCs from biopsies of cancer patients provides us with a new resource to verify this claim by studying early developmental stage phenotypes of cancer and to characterize cancer progression [196]. These observations acquire significant translational potential with the realization that the identification of these developmental stage-specific pathways for cancer resistance/vulnerability could yield major actionable insights for managing tumors and understanding transcriptional regulation.

Ongoing studies progressively elucidate the underlying gene networks in each system, highlighting the similarities and differences. Remarkably, epithelial-to-mesenchymal transition (EMT) is an essential molecular and cellular process involved in neurodevelopmental morphogenesis (synaptic brain plasticity) as well as cancer stem cell plasticity in malignant cancer metastasis progression [197, 198]. EMT is a central process during brain development that affects selected progenitor cells and drives the onset of cellular migrations and subsequent brain morphogenesis, intimately associated with the segregation of homogeneous precursors (neural crest and somites, progenitors of the peripheral nervous system) into distinct fates. Polarity affects stem cell function and allows stem cells to integrate environmental cues from distinct niches in the developing cerebral cortex or in the tumor microenvironment. The crucial role of polarity in stem and progenitor cells is highlighted by the fact that impairment of cell polarity is linked to both neurodevelopmental disorders such as autism spectrum disorders (ASD) as well as cancer cell metastasis [199]. Future research into the biochemical logic of this normal ontogenetic neurodevelopmental process versus pathological conditions such as metastasis–carcinogenesis will further improve our understanding of the paradoxical pathophysiological roles of ADNP in ASD and cancer metastasis [200, 201].

However, cellular heterogeneity in the human brain and the tumor microenvironment complicates the characterization of functional cellular regulatory circuits to form billions of synaptic neuronal connections or controlling cellular migration, cell proliferation as well as programmed cell death. Given the cellular heterogeneity of brain and cancer samples investigated, spatially mapped chromatin analyses at the single-cell level will be especially informative for future studies of neural disorders and cancer development [202]. The recent implementation of advanced single-cell RNA-seq, ChIP-seq, Cut-Tag-seq, DamID, ATAC-seq, GAM, and Hi-C technologies should prove new insights in this regard [203–206]. Development of innovative epigenome editing tools will further enable a functional dissection between altered epigenetic chromatin states and disease outcomes [207, 208].

Variations in the chromatin state of the DNA now reveal micro-level regulations, including heterochromatin nanodomains [209, 210]. Heterochromatin nanodomains are short stretches of ~ 3 to 10 nucleosomes worth of DNA (~ 0.7 to 2 kb, where eukaryotic cells have a characteristic average nucleosome spacing of ~ 190 bp, corresponding to a ~ 45 bp linker). Heterochromatin nanodomains can establish cell type- and development-specific chromatin patterns affecting gene expression programs. Strikingly, heterochromatin nanodomains are determined by DNA sequence-specific binding proteins, including the histone methyltransferases SUV39H1 (KMT1A) and SUV39H2 (KMT1B) that set H3K9me3 marks; methyltransferase GLP (G9a like protein, KMT1D) that forms H3K9me; transcription factor ADNP that recruits the chromatin remodeler CHD4 as well as HP1*β*/*γ* for H3K9me3 mediated gene silencing; chromatin remodeler ATRX that induces the formation of H3K9me3 heterochromatin nanodomains at repeat sequences. These sequence-binding motifs act as nucleation sites and boundaries, cooperative interactions between nucleosomes as well as nucleosome-HP1 interactions. In fact, the number of ADNP motifs per heterochromatin nanodomain is a very good predictor of the HND size. ADNP, like CTCF, is a zinc finger DNA-binding protein, where the ADNP DNA-binding motifs, like CTCF-binding motifs, encompass a set of unusually long sequences (12-mer for ADNP, ~ 19-mer for CTCF), some of which include methylatable CpG sites [39, 65, 211]. A subset of the ADNP DNA-binding motifs closely resembles a subset of the CTCF DNA-binding motifs. Indeed, ADNP, in the ChAHP complex, can compete against CTCF for binding to a subset of sites. Heterochromatin nanodomains typically involve weak CTCF-binding sites, which are often missed with the typical strong CTCF peak-binding detection thresholds of CTCF ChIP-seq data. It has been suggested that weak CTCF sites may be functionally important in defining nanodomain structures, with transient or competitive binding of CTCF and/or ADNP [209, 210]. The effect of ADNP mutations upon heterochromatin nanodomains is unknown and represents an area of future research.

In summary, we have in this extensive review highlighted two and a half decades of ADNP investigations. It clearly highlights the many suggested functions of the *ADNP* gene. While a role in chromatin remodeling is obvious, the relative importance of the various interacting complexes is yet to be determined. This is even more true for its suggested cellular functions. Similarly, though mutations in *ADNP* do result in autism and to play a suggested role in cancer, the mutational mechanism remains to be further investigated. Recent technological developments will now enable us to zoom in on the yet unexplored functions of *ADNP* and their role in disease.

## Data Availability

Not applicable.
